# Treatment of Hypothyroidism and Stress Using Neuro-Emotional Technique (NET): A Case Study

**DOI:** 10.7759/cureus.58231

**Published:** 2024-04-14

**Authors:** Peter Bablis, Ryan R Day, Sophia Bablis, Henry Pollard

**Affiliations:** 1 Department of Maternal and Child Health and Precision Medicine, University Research Institute, Athens, GRC; 2 Department of Integrative and Complementary Medicine, Universal Health, Sydney, AUS; 3 Department of Chiropractic, Universal Health, Sydney, AUS; 4 Department of Psychology, Universal Health, Sydney, AUS; 5 Faculty of Health Sciences, Durban University of Technology, Durban, ZAF

**Keywords:** hashimoto's thyroiditis, mindfulness-based intervention (mbi), case report, adverse childhood experiences (aces), allostatic load, psycho-immune-neuroendocrine (pine) network, neuro-emotional technique (net), hypothyroidism

## Abstract

Hypothyroidism is generally considered an autoimmune condition, and typical medical management involves taking levothyroxine (synthetic thyroid hormone) for life. This case report details the results of a mind-body intervention (MBI) called the Neuro-Emotional Technique (NET) used to treat a 28-year-old Caucasian female presenting with symptoms and bloodwork markers associated with two years of hypothyroidism and a long history of stress. The patient's medical doctor provided a diagnosis of hypothyroidism after blood tests showed that thyroid-stimulating hormone (TSH) levels were high at 6.87 mIU/L (where the acceptable range is 0.40-3.50 mIU/L) and free T4 (FT4) levels were low at 8.6 pmol/L (where the acceptable range is 9.0-19.0 pmol/L). Psychometric tests were completed at baseline and after 12 weeks of treatment to evaluate changes in mental health and emotional well-being. The Adverse Childhood Experiences Questionnaire (ACE-Q) revealed a high degree of childhood trauma that may have predisposed to the underlying autoimmune thyroid dysfunction. At the conclusion of the treatment period, serum thyroid-stimulating hormone (TSH) and free T4 were within normal ranges and psychometric indicators normalized. We hypothesize that these changes may be due to the stress-reducing mechanism of NET and outline possible mechanisms via the Psycho-Immune-Neuroendocrine (PINE) network. The PINE network model asserts that chronic stress acts as a potential driver of pathophysiology that can lead to one or more medical and mental health conditions. While further studies with larger sample sizes are required to establish whether these results could be extrapolated to a wider population, the results of this case suggest that it may be pertinent to consider co-management of subclinical hypothyroidism with a relatively quick and cost-effective MBI such as NET.

## Introduction

Primary hypothyroidism, which makes up 99% of all hypothyroidism cases, occurs when there is inadequate production of thyroid hormones (T4 and T3) for the body's requirements [1], so the pituitary gland increases production of thyroid-stimulating hormone (TSH) through a negative feedback mechanism [2]. Worldwide, the most common cause of all thyroid disorders is iodine deficiency, but in areas of iodine sufficiency, the most common form of primary hypothyroidism is known as Hashimoto's thyroiditis [1,3,4]. Hashimoto's thyroiditis is an autoimmune disorder that causes the immune system to attack the thyroid gland, resulting in swelling, inflammation, and destruction of thyroid cells, disrupting normal thyroid hormone production [5]. As more and more thyroid cells are destroyed, the thyroid gland becomes unable to produce thyroid hormones T3 and T4 in sufficient quantities, and the disease may progress from subclinical to overt hypothyroidism in the absence of appropriate treatment.

Hypothyroidism is estimated to affect up to 5% of the population. Studies in the USA and Europe estimate that a further 5%-7% have undiagnosed thyroid dysfunction [6-8]. Hypothyroidism is associated with poorer quality of life (QoL) and negative health outcomes [9,10], as well as excess mortality of around 50% [11], highlighting the importance of early diagnosis and management of the condition. Remission of hypothyroidism in adults is a rare occurrence [12], estimated to occur at a rate of less than 5% [13,14]. Conventionally considered a permanent and incurable condition, primary hypothyroidism typically requires lifelong hormone therapy with levothyroxine (LT4) or a similar synthetic T4 to normalize TSH levels [14-16]. However, suboptimal dosing is common in clinical practice, and other factors such as poor patient compliance and drug interactions can lead to inadequate treatment [17]. Inadequate pharmacological management may also correlate with poorer mental health status in sufferers [18]. A subset of patients may find that symptoms persist despite undergoing thyroid hormone replacement and normalization of TSH levels [19,20].

Serum biochemistry (often including T3, T4, TSH, and thyroid antibodies) is relied upon for diagnosis because the symptoms and presentations of hypothyroidism can vary wildly. Untreated hypothyroidism can contribute to symptoms as diverse as fatigue, weight gain, constipation, hair loss, cognitive impairment, neuromuscular dysfunction, dyslipidemia, infertility, hypertension, and even death [21-23].

Beyond physical symptoms, hypothyroidism is commonly associated with adverse mental, emotional, relational, social, and work-related outcomes, highlighting the necessity of a biopsychosocial (BPS) approach to treatment [24]. (A detailed description of the BPS approach applied in this case can be found under the heading "Assessment and Examination.") Furthermore, preliminary evidence suggests that scores of 2 or greater on the Adverse Childhood Experiences Questionnaire (ACE-Q) may be associated with up to 70%-80% increase in the risk of hospitalization for autoimmune conditions (including, but not limited to, hypothyroidism [25]). However, care should be taken in implying causation in a study that demonstrates association.

The purpose of this case report is to describe the results of a new stress-relieving mind-body intervention (MBI) called Neuro-Emotional Technique (NET) using outcome measures of serum markers (TSH and free T4 (FT4)) and psychosocial indicators (ACE-Q, Depression, Anxiety, and Stress Scale (DASS-21), Distress and Risk Assessment Method (DRAM), and Short-Form McGill Pain Questionnaire (SF-MPQ)) of hypothyroidism and chronic stress in a 28-year-old Caucasian female.

## Case presentation

The personal information of the patient has been de-identified, and consent was given for the publication of personal health information in print and digital format (without divulging personal identifiers). The patient described in this case report is a 28-year-old Caucasian female presenting with hypothyroidism that had been active and unresolved for two years prior to presenting for treatment at this practice. The patient was comfortable with the diagnosis when it was first presented to her by her general practitioner (GP). She reported, however, that her symptoms gradually continued to worsen. The patient chose to pursue a mind-body solution for her condition as she was averse to consuming medication. It was noted that there was no possible improvement to the patient's condition attributed to lifestyle changes or other treatments as none were reported by the patient.

Patient's medical history

Timeline of the Patient's Health History

During childhood, the patient experienced abuse, neglect, and trauma. (The patient answered "Yes" to questions 1-5 and 8 and 9 on the ACE-Q, representing a total score of 7). In her teen years, her tonsils were removed, she has irregular periods (ongoing up to the time of presentation), and she suffers from severe alcohol abuse. In her early 20s, she was in recovery from alcoholism with continuous sobriety up to the time of presentation. She began to experience ongoing physical symptoms including regular headaches, fatigue and lethargy, ringing in the ears, "brain fog," a constant feeling of tightness in the throat, and muscular tension in the hips. She started seven years of regular psychotherapy to address issues of childhood trauma and abuse; however, her physical health had not improved nor had her symptoms been relieved during this time. From her mid-20s to the present day, her physical symptoms worsened. Her mental health (anxiety and depression) also worsened and became unmanageable.

Prior to presentation at this clinic, the patient's medical doctor diagnosed hypothyroidism based on blood test results showing that TSH levels were high at 6.87 mIU/L (where the acceptable range is 0.40-3.50 mIU/L) and free T4 levels were low at 8.6 pmol/L (where the acceptable range is 9.0-19.0 pmol/L). T3 and thyroid antibodies were not assessed by the patient's medical physician, and reasons for these omissions were not provided at the request of the patient. Ultrasound showed that the thyroid gland was normal in size; however, there was evidence of significant general hyperemia.

Assessment and examination

Based on the work of Engel [26], a biopsychosocial (BPS) approach to assessment and examination was adopted to assess the interplay of biological, psychological, and social factors that may have contributed to this patient's presenting symptomatology [27]. This approach is recognized as being particularly helpful in addressing chronic disorders [28] and was conducted on the initial visit.

Biological Factors

The practitioner performed a postural analysis and physical examination of the spine and musculoskeletal system. This included a range of motion tests, neurological and orthopedic tests, and palpation. Recent blood tests and prior medical diagnoses were reviewed.

Psychological Factors

To assess the patient's viewpoint related to pain, physical symptoms, and psychological well-being, the assessment included the following psychometric tests: the Adverse Childhood Experiences Questionnaire (ACE-Q) [29], the Depression, Anxiety, and Stress Scale (DASS-21) [30], the Distress and Risk Assessment Method (DRAM) [31,32], and the Short-Form McGill Pain Questionnaire (SF-MPQ) [33].

Social Factors

The practitioner took a detailed personal health history, including relevant family health history, and incorporated aspects of the patient's significant social, cultural, and interpersonal life experiences and challenges, as described in the medical history above. The patient's personal decision to not pursue medical management of the hypothyroidism was discussed, as were possible avenues for treatment. The patient's preference was to first try a non-pharmacological treatment before considering conventional pharmacotherapy.

The NET treatment protocol

NET is a 15-step stress-reducing mindfulness-based intervention (MBI) aimed at improving physical and emotional health [34]. The methodology is used to find and remove unresolved stress patterns called neuro-emotional complexes (NECs) [34]. When performing NET, the practitioner utilizes a reliable biofeedback mechanism called a manual muscle test [35,36] to aid in the identification of involuntary physiological responses to specific physical and/or verbal stimuli [37]. NET has been examined in previous case reports [38] and studies [39] as a potential first-line or co-management treatment option for hypothyroidism.

NET draws on aspects of cognitive behavioral psychology as well as diagnostic methods employed in traditional Chinese medicine (TCM) [40] with the goal of reducing or extinguishing classically conditioned painful emotional responses to trauma or stress. NET has been shown in randomized controlled trials (RCTs) to reduce levels of five inflammatory blood markers in the blood commonly associated with disease and increase quality of life indicators for 112 chronic low back pain patients [41]. Monti et al. [42] have also shown reduced activation of the limbic (emotional) brain via the para-hippocampus following the NET treatment for traumatized cancer survivors [42].

Treatment Schedule

The treatment was initially scheduled at two 15-minute sessions per week for four weeks. A review of the patient's progress was conducted on the eighth visit. The patient received a further 12 treatments throughout the following eight weeks, resulting in a total of 20 treatments over 12 weeks. The patient reported no other lifestyle or dietary changes during the treatment period.

Results

The results of the NET treatment show a reduction in psychometric markers of stress and a resolution of the blood markers that led to the initial diagnosis of primary hypothyroidism. Baseline measurements were recorded prior to treatment commencing and again after 12 weeks.

Adverse Childhood Experiences Questionnaire (ACE-Q)

Adverse childhood experiences (ACEs) are an assessment tool designed to measure the severity of adversity in childhood (such as trauma, abuse, and neglect) and its potential to affect a person's physical and mental health in adulthood [29]. ACEs have been found to predispose to a range of chronic health disorders, mental health conditions, risk behaviors, cardiometabolic illnesses, developmental disruption of the brain, and increased healthcare utilization [25,43-46]. It is worthwhile to note that these conditions are commonly present in chiropractic clinics [47,48]; therefore, a trauma-informed approach for all musculoskeletal and allied health practitioners alike is recommended [44,49].

Exposure to ACEs can create chronic neurobiological alterations that predispose to metabolic, behavioral, and cardiovascular morbidity. Due to the vulnerability of the programming of the pathophysiology of the stress response during neurodevelopmental periods in childhood, ACEs have been shown to play a crucial role in the risk of developing a mental or physical health disorder later in life [50]. Another term commonly used in the literature referring to ACEs is early life stress (ELS).

**Figure 1:**
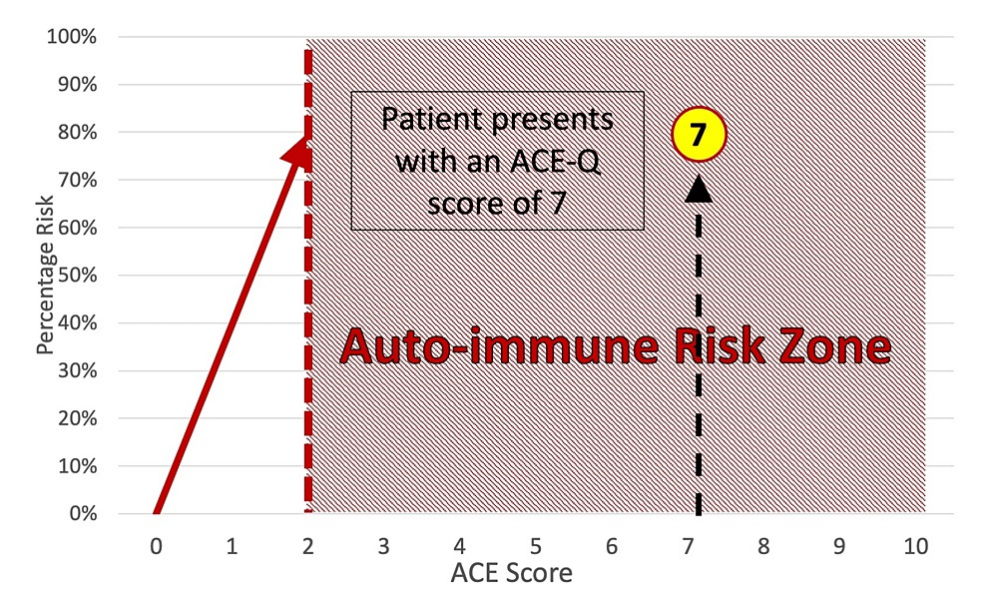
Autoimmune risk zone ACE: adverse childhood experiences, ACE-Q: Adverse Childhood Experiences Questionnaire

The patient in this case report scored 7 out of 10 on the ACE-Q. This score, when correlated with the patient's self-reported high stress levels, symptomatic presentation, and health history, compels consideration of the contribution that ACEs/ELS may have made to the hypothyroidism years later. Research by Dube et al. [25] has shown that an ACE score of just 2 or greater represents a 70%-80% increased risk of experiencing an autoimmune condition in adulthood: what we have called the "autoimmune risk zone" (as depicted in Figure 1). Hypothyroidism is one of many potential autoimmune conditions that may be associated with higher ACE scores, with the incidence of first hospitalization increasing, in a dose-dependent manner, with every increasing number on the ACE-Q [25].

Depression, Anxiety, and Stress Scale (DASS-21)

The Depression, Anxiety, and Stress Scale (DASS-21) is a popular questionnaire for self-assessment of the severity of symptoms of depression, anxiety, and stress [30]. It is regarded for its dependability as a psychometric tool due to exceptional internal consistency and reliability [51]. Increased scores indicate symptoms that are more serious in nature. "Extremely severe" scores indicate that the patient may be dealing with severe psychological symptoms that can seriously impair their general well-being and quality of life.

**Figure 2:**
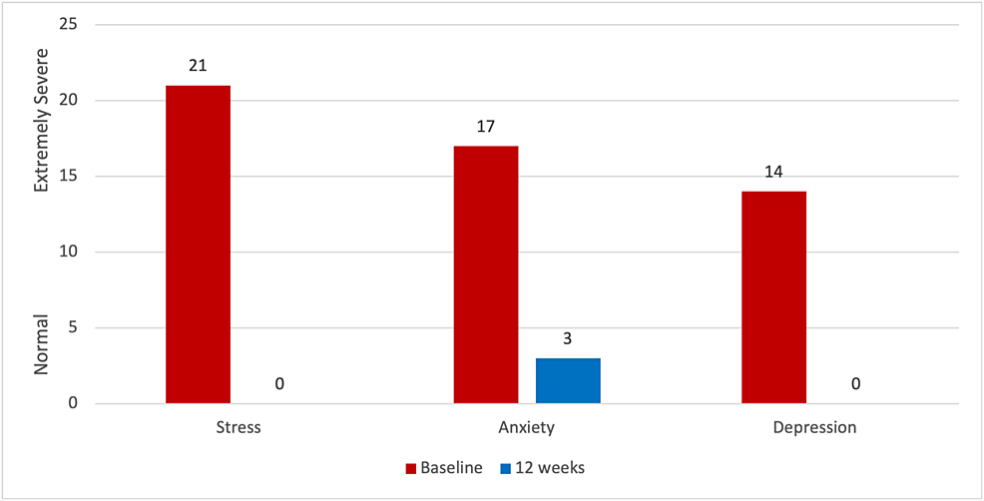
DASS-21 results at baseline and 12 weeks DASS-21: Depression, Anxiety, and Stress Scales

At baseline, the three DASS-21 scores for this patient were "extremely severe" in each category: stress, 21; anxiety, 17; and depression, 14. At the conclusion of the treatment period, the patient's results (as shown in Figure 2) were all within the normal range: stress, 0; anxiety, 3; and depression, 0.

Distress and Risk Assessment Method (DRAM)

The Distress and Risk Assessment Method (DRAM) is a validated measuring tool consisting of two questionnaires, the Modified Zung Depression Index (MZDI) [52] and the Modified Somatic Perception Questionnaire (MSPQ) [53]. The MZDI is designed to give a quantitative measure of a person's level of depression, while the MSPQ is a measure of somatic awareness and anxiety (distress). The combined results of the MZDI and the MSPQ are tallied to provide a DRAM score. A high score is regarded as a poor predictor of treatment success [31,32].

The DRAM gives a straightforward way to categorize patients into three groups (those who exhibit no psychological distress, those who are likely to experience substantial psychological distress, and those who are clearly distressed), resulting in the classification of four different patient types: normal, at risk, distressed-depressive, and distressed-somatic.

**Figure 3:**
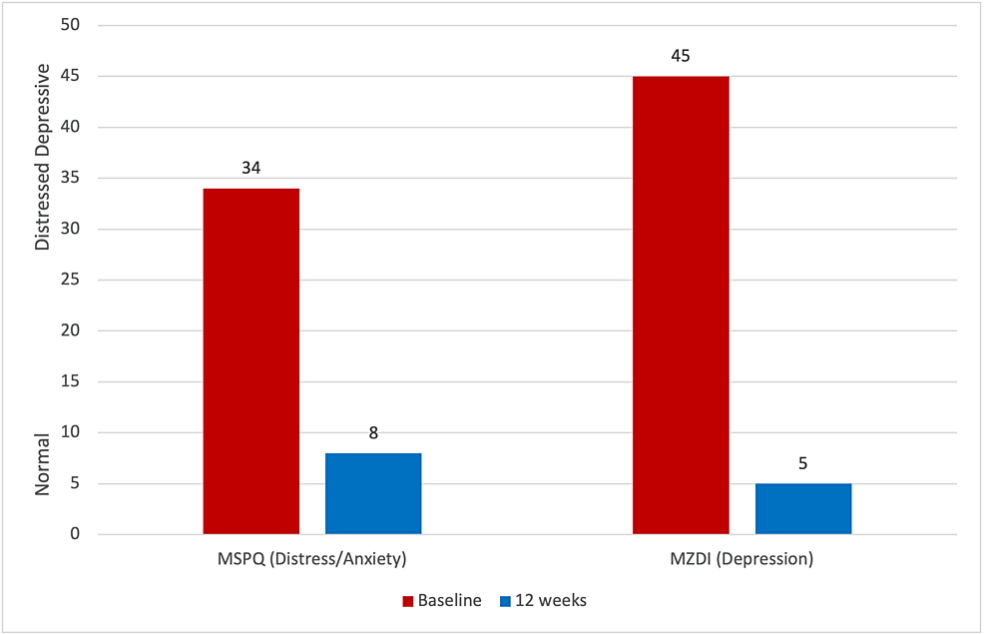
DRAM results at baseline and 12 weeks DRAM: Distress and Risk Assessment Method, MSPQ: Modified Somatic Perception Questionnaire, MZDI: Modified Zung Depression Index

The patient completed the DRAM at the commencement of the treatment program and again after 12 weeks (20 treatments). The patient's initial scores were an MSPQ (indicating distress) of 26 and an MZDI (indicating depression) of 34, resulting in a classification of "distressed-depressive." As shown in Figure 3, after 12 weeks, these scores dropped to 8 and 5, respectively, resulting in a classification of "normal."

Short-Form McGill Pain Questionnaire (SF-MPQ)

The Short-Form McGill Pain Questionnaire (SF-MPQ) is a reliable and valid assessment of a person's intensity and frequency of physical discomfort [33]. It includes four primary components: the Present Pain Intensity (PPI) scale, the Visual Analog Scale (VAS), 11 sensory (physical) pain descriptors, and four affective (emotional) pain descriptors that can be scored on an intensity scale where 0 = none, 1 = mild, 2 = moderate, and 3 = severe.

**Figure 4:**
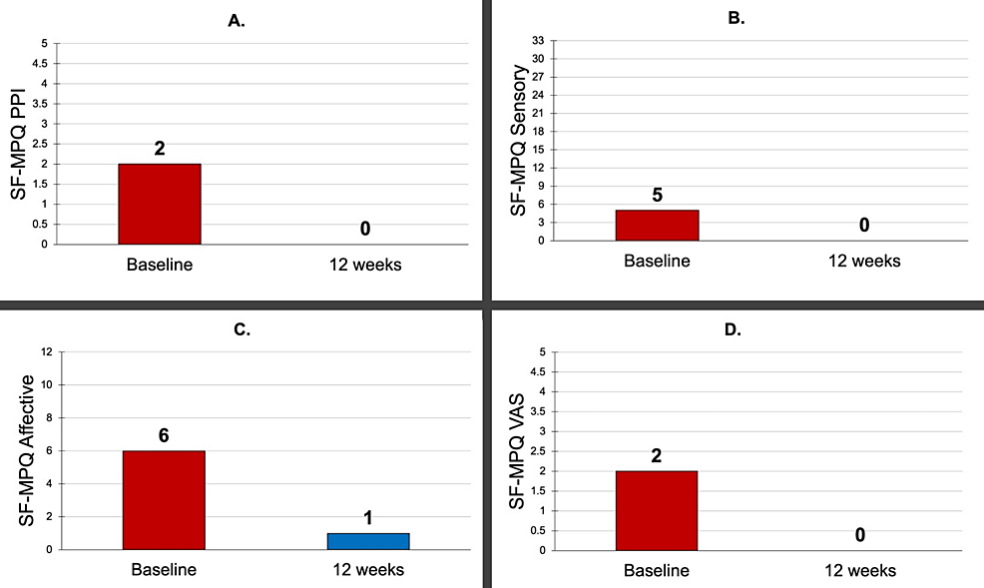
SF-MPQ results at baseline and 12 weeks A: SF-MPQ PPI, B: SF-MPQ Sensory (physical pain), C: SF-MPQ Affective (emotional pain), D: SF-MPQ VAS SF-MPQ: Short-Form McGill Pain Questionnaire, PPI: Present Pain Intensity, VAS: Visual Analog Scale

The results of the SF-MPQ showed a 100% decrease in PPI from a baseline score of 2/5 to 0/5 at 12 weeks. Sensory (physical) pain was reduced by 100% from 5/33 to 0/33 at 12 weeks. The affective (emotional) pain score dropped more than 80% from 6/12 to 1/12, and the visual analog scale (VAS) pain rating fell 100% from 2/5 to 0/5 at 12 weeks. Results are shown in Figure 4.

Thyroid Blood Markers

**Figure 5:**
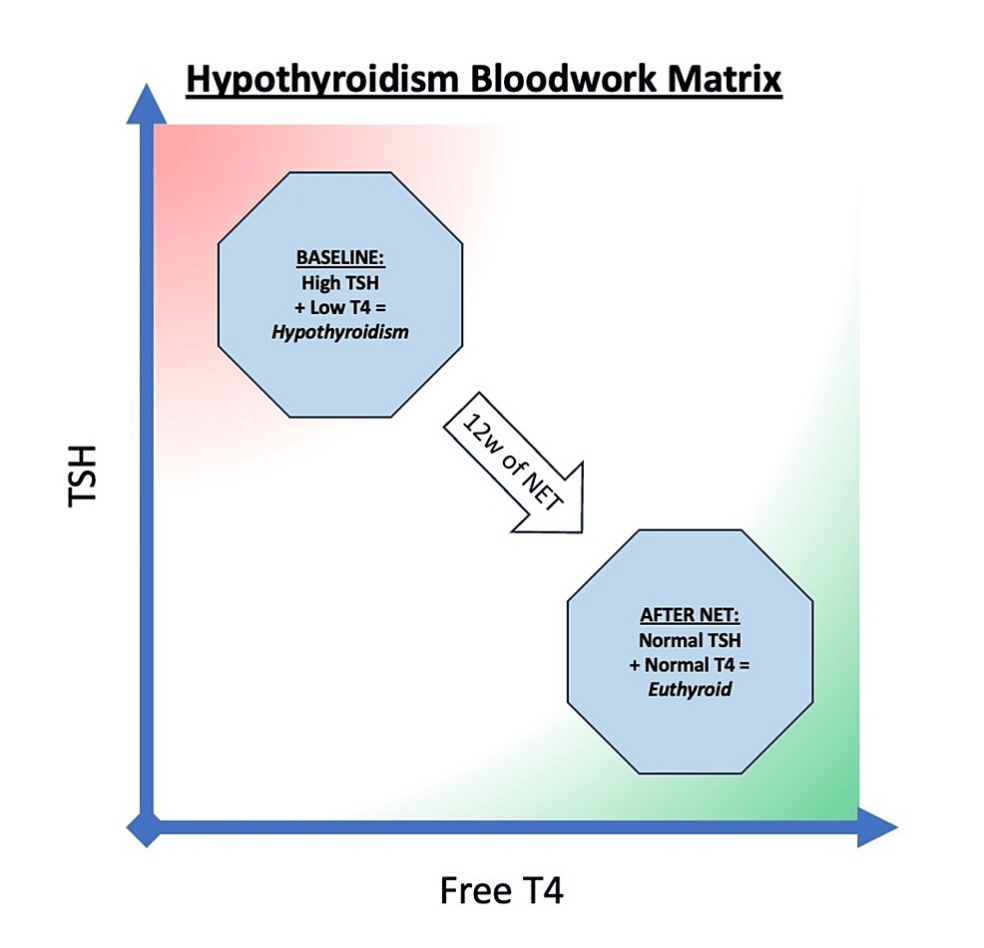
Hypothyroidism bloodwork changes during 12 weeks of NET treatment High TSH and low FT4 are the blood test markers commonly relied upon for diagnosing hypothyroidism. TSH: thyroid-stimulating hormone, FT4: free T4, euthyroid: blood markers indicate normal thyroid function

Overt primary hypothyroidism is biochemically diagnosed when serum thyroid-stimulating hormone (TSH) is above the reference range and free thyroxine (T4) levels drop below the reference range [54]. When these thresholds are exceeded, standard medical treatment involves the prescription of levothyroxine (LT4), one of the most frequently used medications globally [6,55,56]. The patient's personal preference was to seek a non-pharmaceutical, mind-body treatment to address the hypothyroidism. Blood markers were consistent with primary hypothyroidism prior to the NET treatment, and at the conclusion of the treatment period, these blood markers were within the normal ranges, as seen in Table 1 and Figure 5.

**Table 1:** Thyroid blood test results at baseline and 12 weeks TSH and FT4 are commonly relied upon for diagnosing primary hypothyroidism. TSH: thyroid-stimulating hormone, FT4: free T4

Timeline	TSH (acceptable range: 0.40-3.50 mIU/L)	FT4 (acceptable range: 9.0-19.0 pmol/L)	Comment
Baseline	6.87 (high)	8.6 (low)	Indicative of primary hypothyroidism
12 weeks	0.95	14.4	Euthyroid (i.e., markers are within normal ranges)

Notably, a substantial proportion of patients receiving the typical pharmacological treatment report persistent complaints despite reaching the biochemical therapy targets [21,57,58]. In addition, prescription levothyroxine has been shown to diminish the quality of life in a subset of patients compared to controls [19]. This may be due to an overreliance on reaching biochemical TSH targets rather than focusing on relieving specific symptoms, ill-defined TSH reference ranges, or confounding factors (such as comorbidities or obesity [59]) that complicate the diagnosis of thyroid dysfunction by bloodwork alone [60].

## Discussion

This case report highlights a patient who experienced stabilization of her hypothyroidism blood markers without pharmacotherapy after 12 weeks (20 sessions) of management with NET. This patient's anxiety and distress levels improved during the 12-week intervention period as evidenced by the results of the psychometric tests performed before and after the treatment.

While randomized controlled trials are needed to further assess the effectiveness of the treatment before confidently assuming causality, NET has been previously shown to correlate with normalized blood markers in other cases of hypothyroidism [38]. Furthermore, serum inflammatory markers have been shown to improve in an RCT studying the effect of NET on a cohort of chronic low back pain patients [41]. To our knowledge, however, this case report is the first to include ACEs as an assessment tool, as well as show improvements in psychometric indicators before and after NET treatment of patients with hypothyroidism. Due to the well-established idea that prolonged or excessive stress exposure may exceed the individual's natural adaptive capacity and cause lasting changes to the stress response system [50,61], we hypothesize that the mechanism of action of NET may be due to the relief or resolution of long-held psychosomatic stressors (such as ACEs and ELS) that initially contributed to the arising of the autoimmune condition.

This is not an entirely new idea, but one that may be nearly forgotten in the almost exclusive reliance upon pharmacological treatments for hypothyroidism. Thyroid dysfunction was first identified in 1825 and was attributed to traumatic fear [62,63]. Later, early scientific reviews of more than 3,000 patients with thyrotoxicosis found that 85% had a clear history of traumatic stress [64]. Changes in thyroid function due to trauma, stress, and psychological disturbances have long been recognized in both human and animal studies [65-68]. A large longitudinal study of nearly 46,000 females identified a dose-dependent relationship between post-traumatic stress disorder (PTSD) and hypothyroidism where the greater number of PTSD symptoms identified correlated with a higher incidence of hypothyroidism [69].

Another study has identified that females who were exposed in childhood to moderate or severe abuse or neglect were found to be 4-7 times more susceptible to subclinical hypothyroidism (high TSH levels and normal free T4 levels) during pregnancy [70]. Whether by compromised thyroid hormone production or increases in anti-thyroid antibodies crossing the placental barrier [71,72], fetal development can be deleteriously affected by maternal thyroid dysfunction. Studies showing an increased risk of neurodevelopmental disorders and adverse effects on a child's brain and cognitive development [73], higher association with attention deficit hyperactivity disorder (ADHD) [74,75], lower intelligence quotient (IQ)/cognitive performance [76,77], and lower hippocampal volume in their children [78] all highlight the potential downstream consequences of even mild imbalances in thyroid hormones. These findings lend weight to the concept of "intergenerational trauma," suggesting that maternal exposure to childhood maltreatment (ACEs) may influence gestational biology during intrauterine development or even prior to conception [79].

The Psycho-Immune-Neuroendocrine (PINE) network model [80] asserts that chronic stress (including ACEs/ELS, unresolved individual or intergenerational trauma, and cumulative microstressors) acts as a potential driver of pathophysiology that can lead to one or more medical and mental health conditions [81,82]. Moog et al. [70] proposed three possible biochemical pathways that could explain how childhood ACEs/ELS may result in hypothyroidism. A first possibility is that, because stress alters the function of the immune system through the hypothalamic-pituitary-thyroid (HPT) axis [83], this may result in epigenetic influences that "produce a permanent or long-lasting shift in the homeostatic setpoint of the HPT axis, which may then increase susceptibility for developing thyroid dysfunction later in life [71]." A traumatic experience of abuse or neglect may create a sense of defensiveness or helplessness, leading to an adaptive "survival" strategy of immobilization, withdrawal, and energy conservation, which may in turn be mediated by a long-term lowering of thyroid function [70].

A second possible mechanism is the long-term influence the dysfunctional thyroid can exert on the integrity of the hypothalamic-pituitary-adrenal (HPA) axis [84], a stress-regulating system that is strongly interconnected with the HPT axis. Hypoactivity of the HPA axis (i.e., lower circulating levels of cortisol or blunted cortisol stress response) has been associated with both childhood trauma [85-90] and resultant enduring health consequences, such as hypothyroidism [50].

A third possibility is that exposure to traumatic experiences or maltreatment in childhood may affect thyroid function via its long-term effects on inflammation and immune function. ACE scores of 4 or more have been found to strongly correlate with autoimmune conditions in adulthood [25], highlighting possible links between stressful events in childhood, inflammation, and immunity. A meta-analysis conducted by Baumeister et al. [91] demonstrated a robust association between childhood trauma and elevated peripheral blood pro-inflammatory cytokines (C-reactive protein (CRP), interleukin-6 (IL-6), and tumor necrosis factor-α (TNF-α)) in adulthood. Inflammatory cytokines subsequently inhibit the production and secretion of thyroid hormones [92,93] and are known to be directly associated with autoimmune thyroiditis [94,95]. For example, a Th1-dominant pattern of elevated cytokines (suggesting cellular immunity) leads to autoimmune thyroiditis [96] and accelerates its progression by increasing the proliferation of TH17 lymphocytes [94].

While these proposed biochemical pathways are still speculative and require further investigation, there is growing evidence that patients receiving NET demonstrate beneficial changes in immune markers when traumatic and emotional stress is relieved from the body. In a randomized controlled trial, NET was shown to reduce inflammatory cytokines (CRP, TNF-α, IL-1, IL-6, and IL-10) in patients whose levels were above normal ranges after four weeks of treatment with lasting benefits continuing at six-month follow-up after the treatment period. These results accompanied improvements in pain, disability, and quality of life indicators for the treatment group, but not for those who received the control intervention [41]. It is possible that these immune-based, stress-relieving effects of NET might help to reduce allostatic load, thereby possibly restoring function and capacity to the HPA and HPT axes. If this theory can be proven with future studies, it may be shown that NET supports the body's stress response system in a process of recalibration toward a more homeostatic state of bioregulatory function.

NET Compared to Other Treatment Modalities

Conventional pharmacological treatment with levothyroxine (LT4) as a lifelong monotherapy has been the first-line approach to patients with hypothyroidism for approximately seven decades [97] and continues to be regarded as the preferred methodology in recent guidelines [98]. It is considered to reduce symptoms in most patients with a "favorable side effect profile," having the benefits of easy administration, relatively low cost, and good intestinal absorption [99]. A proportion of patients, however, find LT4 to be non-efficacious and unsatisfactory [19,99]. Furthermore, LT4 therapy may be frequently associated with both overtreatment and undertreatment with TSH levels shown in one study to be either too high or too low in up to 57% of older patients [100]. Potentially serious side effects can result. For example, when TSH is over-suppressed, there are increased risks of low bone density and fractures [101], higher hazard ratios for hospitalization and death due to cardiovascular disease [102], and three times increased risk of atrial fibrillation in older persons [103]. Undertreatment, whether due to poor compliance or inadequate monitoring, may give rise to the same comorbidities as the disease such as cardiovascular atherosclerosis [104], congestive heart failure [105], reversible cardiomyopathy [106], and dyslipidemia [107].

Studies in Sweden and Taiwan found a correlation between LT4 and increased cancer risk, especially in females [108,109]. In a Korean nationwide cohort study, overt hypothyroidism was correlated with "significantly higher" all-cause mortality in LT4-treated hypothyroid patients compared to controls [110]. Although LT4 therapy does restore serum thyroxine levels to normal ranges in a portion of patients with overt hypothyroidism, recent investigations indicate that not all thyroid metabolic markers (such as serum low-density lipoprotein (LDL) and total cholesterol) are normalized [111,112]. Health-related quality of life (QoL) scores improve with small to moderate effect sizes up to six months after commencing treatment with LT4 [113]; however, studies report impaired psychological well-being [112,114] and cognitive functioning [115] in euthyroid patients on LT4, revealing that patient QoL does not always correlate with improvements in TSH and/or FT4 blood markers alone [116]. Notwithstanding, LT4 monotherapy continues to be regarded as safe and effective [117] and the gold standard treatment (approved by the Food and Drug Administration (FDA) and the American Thyroid Association [118]) for overt hypothyroidism [99,119]. While some patients receiving conventional pharmacotherapy for overt hypothyroidism experience symptomatic relief, adverse outcome risks may remain even when serum markers of thyroid dysfunction are normalized. For a subset of hypothyroid patients, therefore, it may be appropriate to consider co-management with non-pharmacological treatment modalities, such as NET or other mind-body interventions (MBIs).

How does NET compare to other MBIs for the treatment of primary hypothyroidism? Yoga is one well-known MBI that is being explored as a method of stress reduction [120] and as a potential treatment for endocrine disorders [121]. Preliminary findings of "general yoga" on hypothyroidism in 22 females resulted in TSH levels "moderately" reducing by 9.72% across the group after a full six months of practicing yoga for one hour per day, four days a week (approximately 104 hours) [122]. In comparison, the patient in this case report received 20 NET treatments of 15-minute duration each (totaling approximately five hours of therapeutic intervention) with normalization of hypothyroid blood markers T4 and TSH to within reference ranges, representing significant changes in a much shorter intervention time.

There are both strengths and weaknesses associated with this case report. A weakness of this case report is that the treatment was performed by a single practitioner on a single patient, and therefore, it is possible that the effectiveness of the delivery of the technique is to some degree dependent on the operator. It is possible that NET may not be suitable for all hypothyroidism patients and that the results experienced by the patient in this case report may be unique. While the patient likely represented a typical case of hypothyroidism based on symptoms and previous medical diagnosis, it is possible that an uncommon variant of primary hypothyroidism may have been present. The serum and psychometric tests were only measured immediately before and after the treatment period, so results cannot be generalized for any potential medium- or long-term outcomes of the NET treatment. The influence of the PINE network on hypothyroidism is a hypothesis generated by this case study that requires further research and, as with any single-person case study, natural progression of the disease or the placebo effect cannot be disregarded as possible explanations of the results demonstrated. Until further research is conducted, the results cannot be generalized for other practitioners or for a wider population of hypothyroidism sufferers. A strength of this case report is that the pre- and post-treatment outcome measures could help to establish protocols for larger RCTs that may provide more definitive answers about whether the NET treatment of primary hypothyroidism generates reliable and consistent results.

Hypothyroidism is associated with high healthcare and economic costs due to the prevalence of comorbid conditions [123], decreased quality of life [124-126], decreased productivity because of the increased number of days on sick leave [127], and increased mortality [11]. A US study matching 800,000 hypothyroidism patients with healthy control subjects found that hypothyroidism was associated with more than double all-cause healthcare costs (partially due to comorbidities), along with higher disability and absenteeism costs [128]. Lifelong costs associated with pharmacotherapeutic management of hypothyroidism are estimated to range from $460 to $2,555 per year over a patient's lifetime [128]. In this case report, we have shown normalization of hypothyroidism blood markers with 12 weeks of NET treatment, with previous cases reported by the same author showing similar results in eight weeks in one instance, and four treatments in another [38]. Another paper by the first author examined the results of an RCT and found NET treatment to demonstrate endurance, with implications for cost-savings, due to the positive reduction of inflammatory markers even six months after the completion of just four weeks of NET treatment [129]. If NET can be shown in further large-cohort studies to produce similar changes in hypothyroid blood markers and associated psychometric indicators, it may be considered as a relatively fast-acting and economical non-pharmacological treatment option for certain subtypes of hypothyroidism sufferers.

## Conclusions

This case report suggests that a stress-relieving MBI such as NET might address aspects of the biopsychosocial factors and PINE network elements that potentially contribute to some forms of hypothyroidism. If the results of this case report could be reproduced in longitudinal randomized controlled trials with a greater number of patients and practitioners involved and over longer periods of time (6, 12, or 24 months), this intervention might be shown to be an effective treatment option for some hypothyroidism patients. Further studies testing the hypothetical biochemical mechanisms and longer-term efficacy of the NET treatment, including broader patient-reported outcomes, are highly recommended to determine whether this mind-body intervention might help hypothyroidism sufferers to experience improved outcomes and a better quality of life.
